# Supplementary material for: Structural and functional analysis of the minimal orthomyxovirus-like polymerase of Tilapia Lake Virus from the highly diverged Amnoonviridae family
Source: Nat Commun. 2023 Dec 9;14:8145. doi: 10.1038/s41467-023-44044-x (PMC10709604; doi:10.1038/s41467-023-44044-x)
Supplement: Supplementary file 1 — Supplementary Information [file 41467_2023_44044_MOESM1_ESM.pdf]

SUPPLEMENTARY FIGURE 1

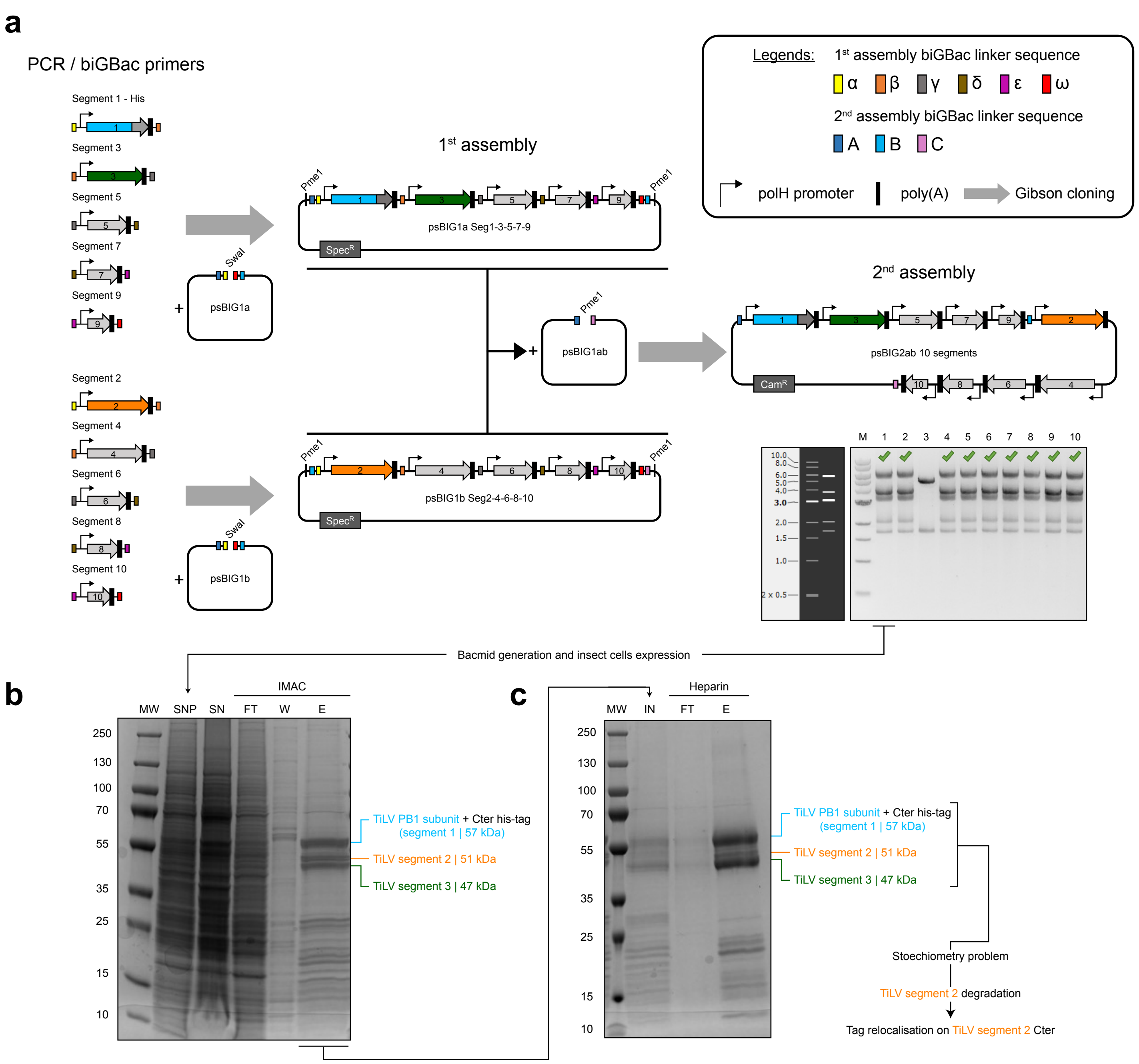

Supplementary Fig. 1. Molecular biology and biochemistry strategies applied to find other segment proteins interacting with TiLV segment 1.
a. Strategy applied to clone and express the ten TiLV viral proteins from one plasmid. TiLV segment 1 is coloured in blue, segment 2 in orange and segment 3 in green. Symbols are defined at the top right. At the bottom right, the agarose gel represents a screen by digestion of ten colonies for selecting a correct psBIG2ab plasmid. A simulation of the digestion is shown to the left of the gel.
b. SDS-PAGE analysis of TiLV polymerase following an initial immobilized metal affinity chromatography (IMAC) purification step, with PB1 subunit bearing a C-terminal (Cter) poly-histidine purification tag (his-tag). The molecular ladder (MW) is on the left. “SNP” corresponds to the total fraction. “SN” to the soluble fraction. “FT” to the flow through. “W” to the wash. “E” to the elution. Deduced TiLV segments are annotated based on their molecular weight.
c. SDS-PAGE analysis of TiLV polymerase following a second heparin affinity purification step, with PB1 subunit bearing a C-terminal (Cter) poly-histidine purification tag (his-tag). “IN” corresponds to the input. “FT” to the flow through. “E” to the elution. Deduced TiLV segments are annotated based on their molecular weight. Stoichiometry and degradation problems of TiLV segment 2 are visible necessitating the relocation of the his-tag to the C-terminus (Cter) of segment 2.

SUPPLEMENTARY FIGURE 2

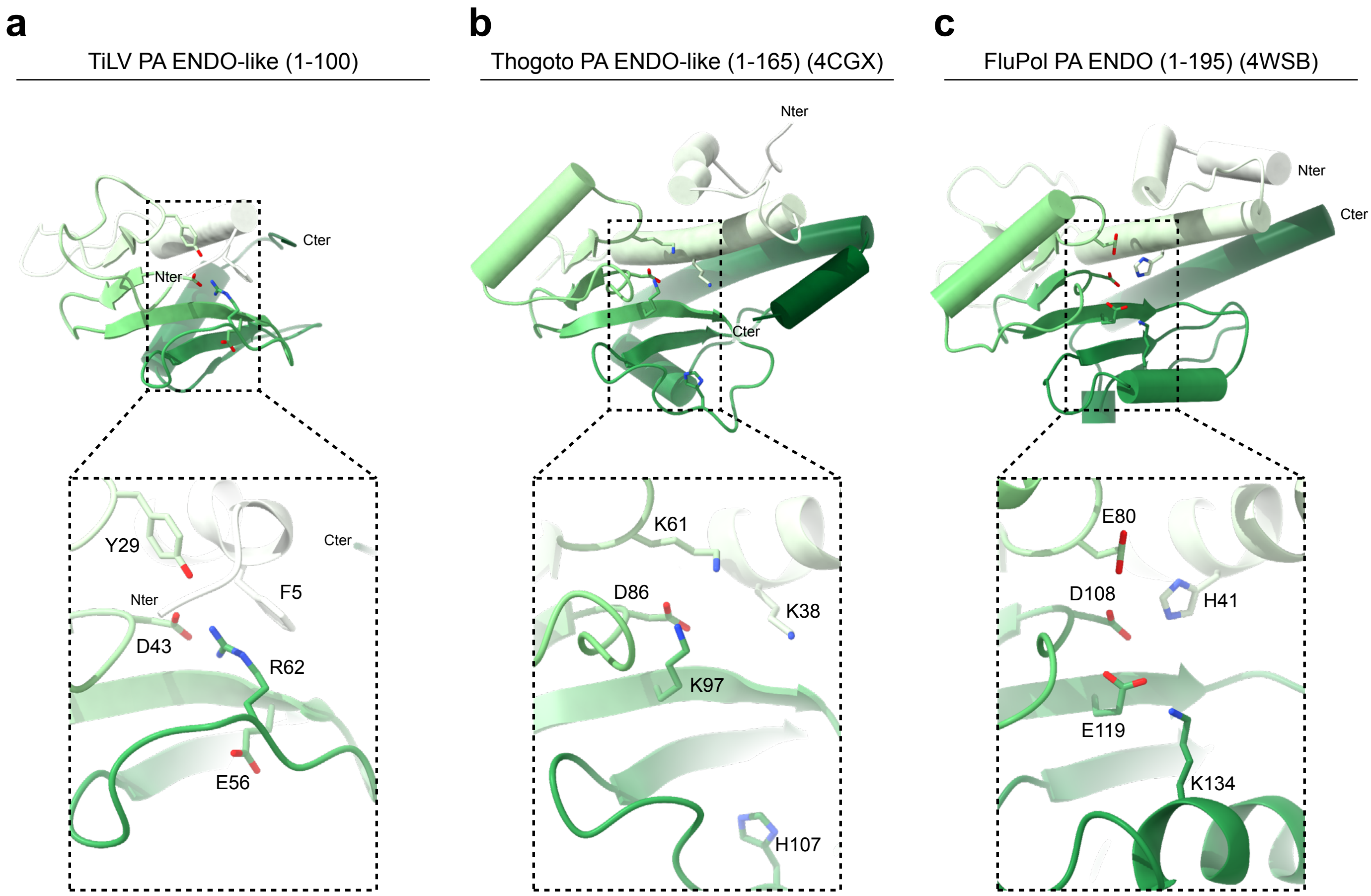

Supplementary Fig. 2. Comparison of TiLV, Thogoto and influenza polymerase PA-N domains.
PA ENDO or ENDO-like domains of (a) TiLV, (b) Thogoto and (c) influenza polymerases. Domains are aligned based on their conserved β-sheets, and coloured from the N-terminus (Nter; light green) to the C-terminus (Cter; dark green). Overall and close-up views of each catalytic (or putative) active site are shown, and corresponding residues are displayed. PDB IDs are indicated.

SUPPLEMENTARY FIGURE 3

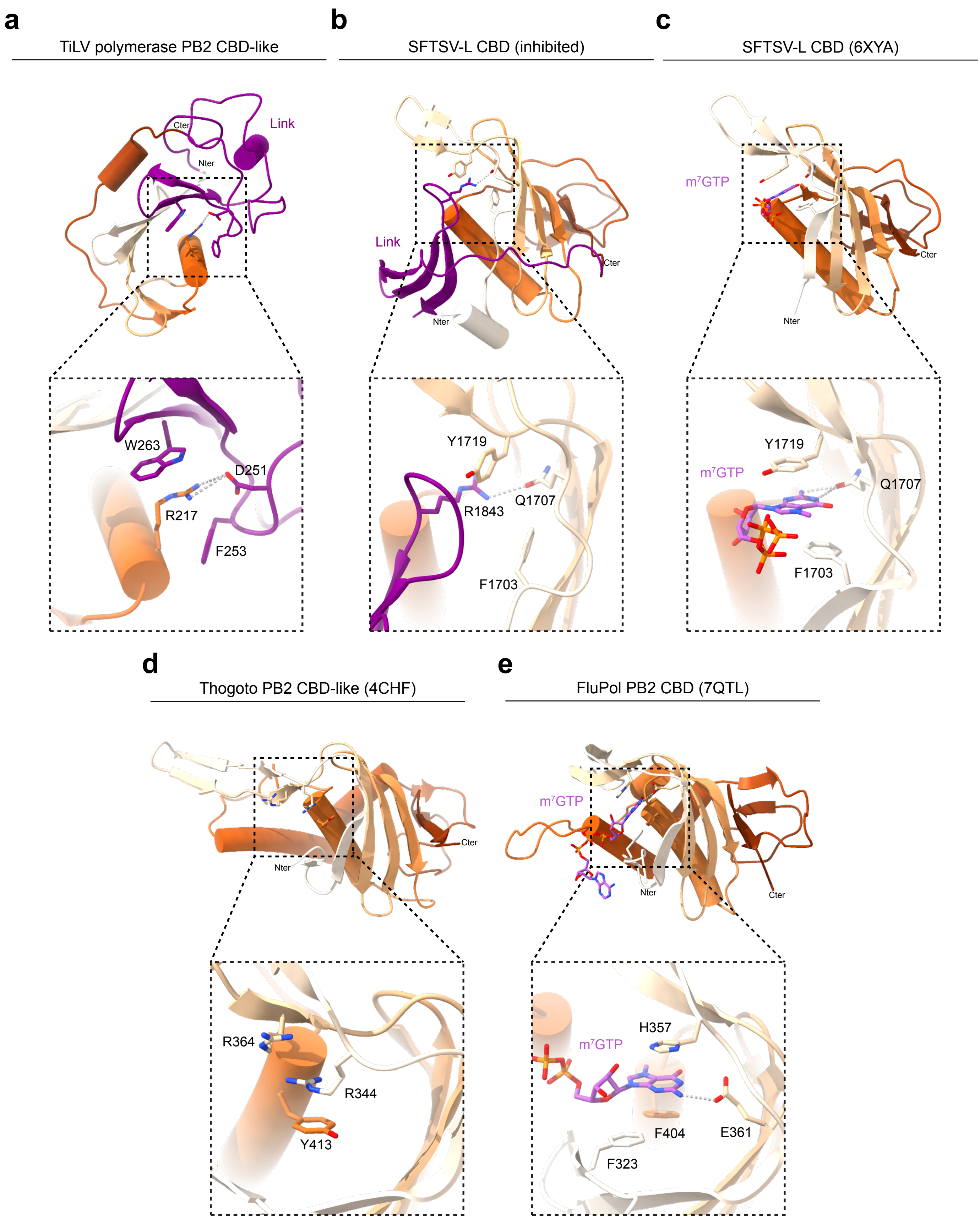

Supplementary Fig. 3. Comparison of TiLV, SFTSV, Thogoto and influenza polymerase PB2 cap-binding domains.
PB2 CBD or CBD-like domains are shown for (a) TiLV polymerase, (b) SFTSV-L (inhibited), (c) SFTSV L (cap-bound), (d) Thogoto and (e) influenza polymerases. Domains were aligned based on their cap-binding site and are coloured from the N- (Nter; light orange) to C-terminus (Cter; dark orange). TiLV polymerase and SFTSV-L PB2 link domains are coloured in magenta. m^{7}GTP atoms are displayed as spheres (overall view) or sticks (zoom view) and coloured in purple. Close-up views of each cap-binding (or putative) site are shown, and corresponding residues are displayed. Hydrogen bonds are represented as grey dotted lines. PDB IDs are indicated.

SUPPLEMENTARY FIGURE 4

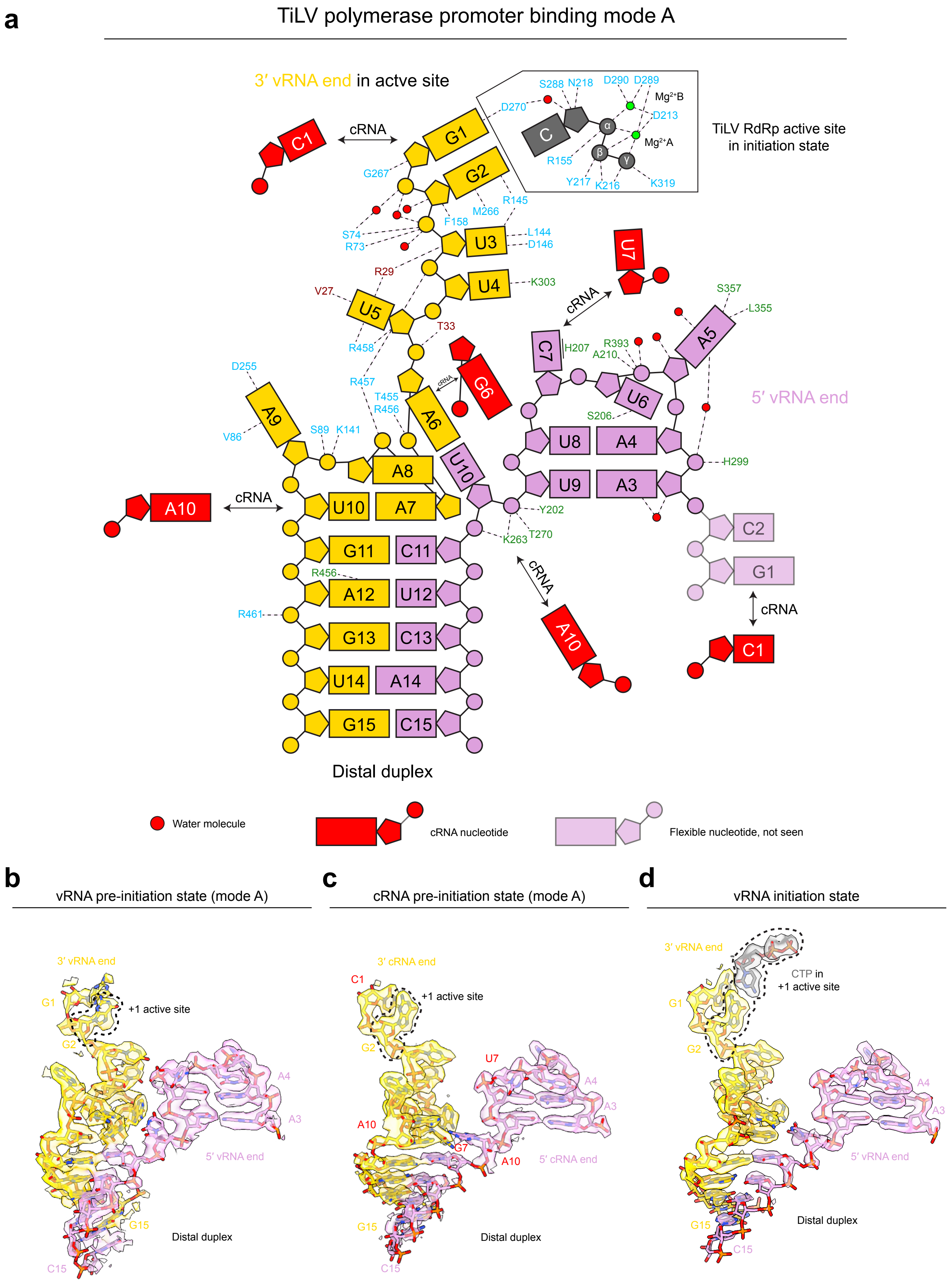

Supplementary Fig. 4. TiLV polymerase promoter binding mode A.

a. Schematic summary of all the interactions between TiLV polymerase and the promoter bound in mode A. The 5' and 3' vRNA ends are respectively in pink and gold. Nucleotide differences between vRNA to cRNA are annotated in red. Flexible nucleotides are in transparent. Water molecules are red spheres and magnesium ions (Mg2+) green spheres. The 3' end nucleotides are numbered from the 3' to 5'. Interacting residues are coloured according to subunit, green for PA, blue for PB1 and dark red for PB2-N. In mode A, the 3' end is in the RdRp active site. In the vRNA initiation state, CTP (dark grey) is in the position +1 of the active site.

b. Extracted RNA density from TiLV polymerase in vRNA pre-initiation state (mode A) cryo-EM map. The +1 active site position is indicated by a dotted line.

c. Extracted RNA density from TiLV polymerase in cRNA pre-initiation state (mode A) cryo-EM map. The v to cRNA nucleotide differences are labelled in red. The +1 position is indicated by a dotted line.

d. Extracted RNA density from TiLV polymerase in vRNA initiation state cryo-EM map. The +1 position is indicated by a dotted line, which encompasses the incoming CTP.

SUPPLEMENTARY FIGURE 5

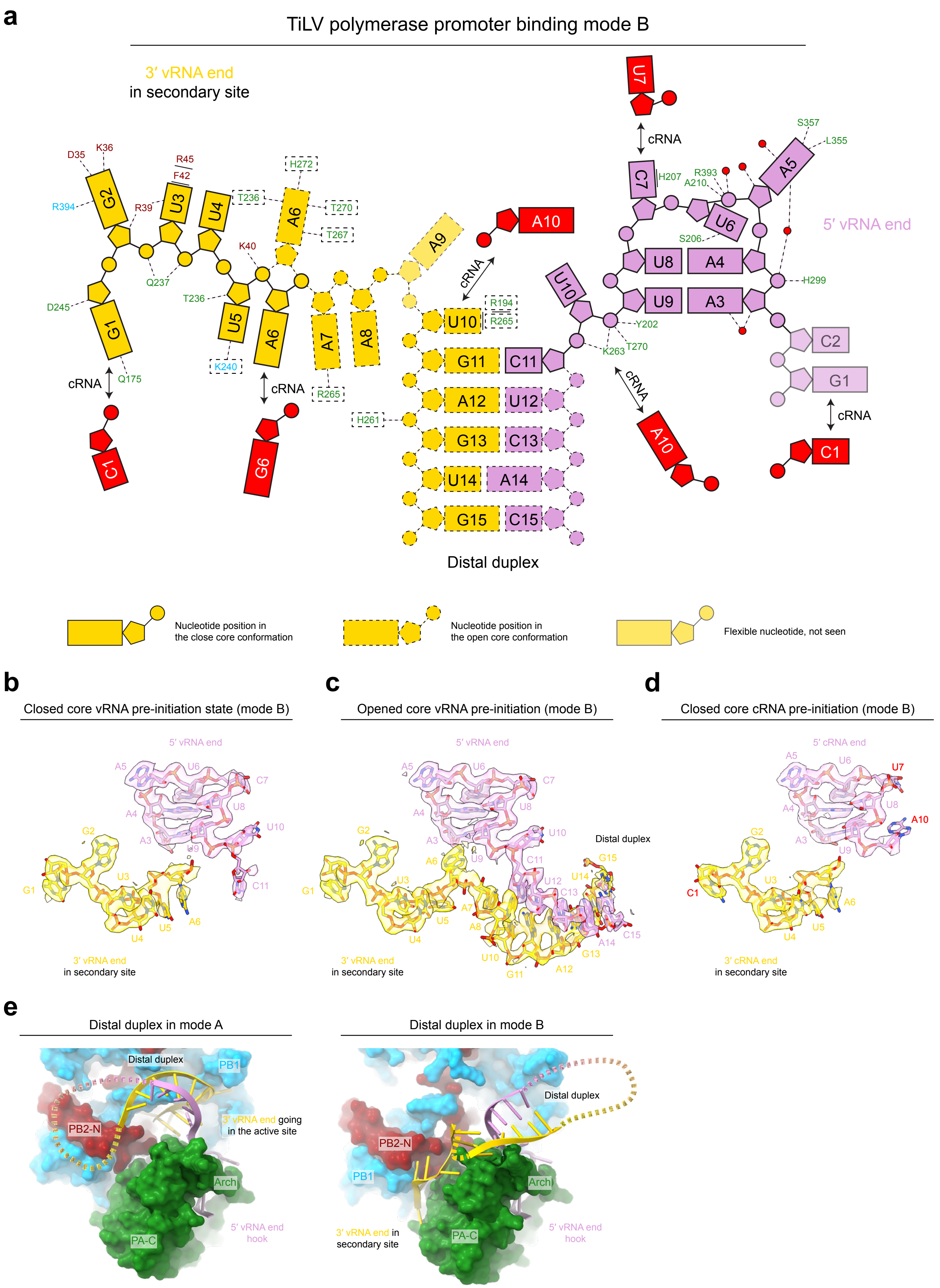

Supplementary Fig. 5. TiLV polymerase promoter binding mode B.

a. Schematic summary of all the interactions between TiLV polymerase and the promoter bound in mode B. The 5' and 3' vRNA ends are respectively in pink and gold. Nucleotide differences between vRNA to cRNA are annotated in red. Flexible nucleotides are transparent. Water molecules are shown as red spheres. The 3' end nucleotides are numbered from the 3' to 5'. Interacting residues are coloured according to subunit, green for PA, blue for PB1 and dark red for PB2-N. In the closed core conformation, the 3' end nts A7-G15 are not visible, whereas in the open core conformation, they are visible and interact with specific residues that are surrounded by dotted rectangles.

b. Extracted RNA density from TiLV polymerase in vRNA pre-initiation state (mode B, close core) cryo-EM map.

c. Extracted RNA density from TiLV polymerase in vRNA pre-initiation state (mode B, open core) cryo-EM map.

d. Extracted RNA density from TiLV polymerase in cRNA pre-initiation state (mode B, close core) cryo-EM map. The v to cRNA nucleotide differences are coloured in red.

e. Comparison of the distal duplex orientation in mode A and B. TiLV polymerase PA, PB1 and PB2 subunits are shown as surfaces and respectively coloured in green, light blue and red. The 5' and 3' vRNA ends are respectively in pink and gold. Flexible nucleotides are represented as dotted lines.

SUPPLEMENTARY FIGURE 6

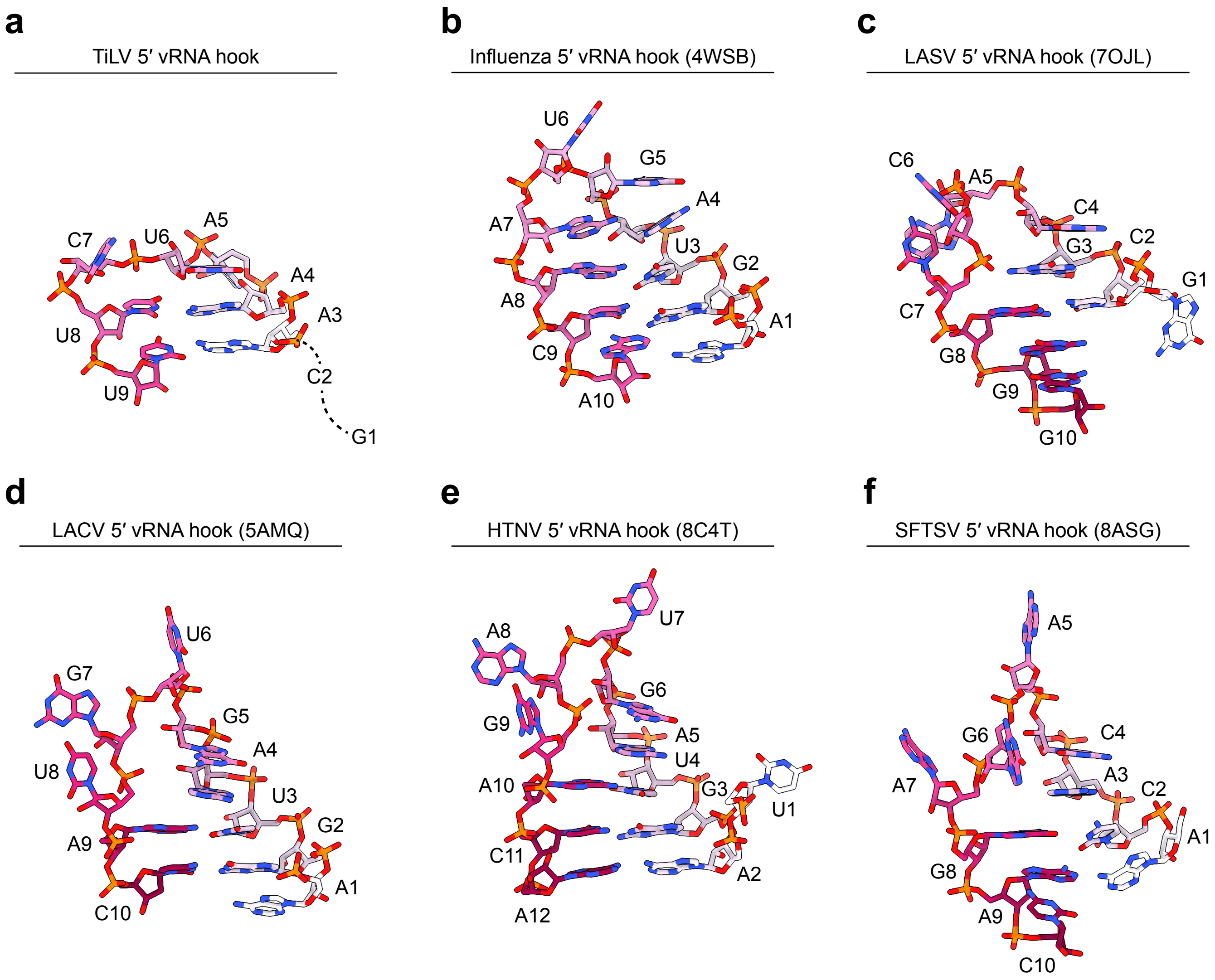

Supplementary Fig. 6. Comparison of 5' hook structures from viral polymerases belonging to the Articulavirales and the Bunyavirales orders. 5' vRNA hooks are shown for (a) TILV, (b) influenza, (c) Lassa virus (LASV), (d) La Crosse virus (LACV), (e) Hantaan virus (HTNV) and (f) severe fever with thrombocytopenia syndrome virus (SFTSV). Nucleotides are numbered and sequentially coloured from the 5' end (light pink) to the 3' end (dark pink). For TiLV, flexible nucleotides are indicated by a dotted line. PDB IDs are indicated.

SUPPLEMENTARY FIGURE 7

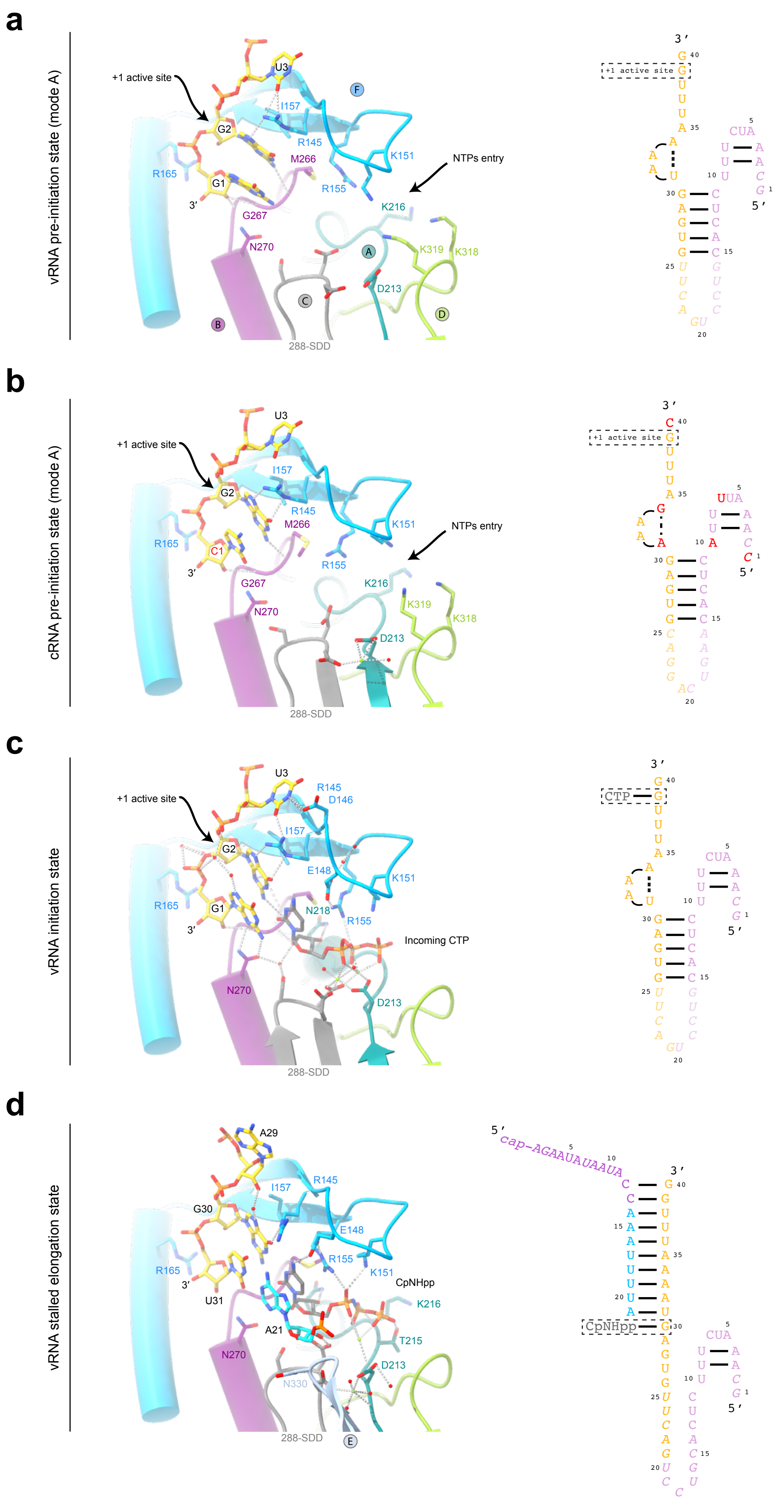

Supplementary Fig. 7. TiLV polymerase, from pre-initiation to elongation state focussing on the active site.

a. Left: TiLV polymerase active site in vRNA pre-initiation state mode A. The 3' vRNA end is coloured in gold and nucleotides are numbered from the 3' to 5', with G1 and G2 being in the respective -1/+1 active site positions. RdRp motifs A, B, C, D, and F are respectively coloured in dark turquoise, purple, grey, light green, and blue. Key residues are displayed. Hydrogen bonds are grey dotted lines. Arrows indicate the +1 active site and the NTP entry channel. Right: schematic of the overall RNA conformation with the +1 active site position indicated with a dotted rectangle. Flexible nucleotides are in italic.

b. Left: TiLV polymerase active site in cRNA pre-initiation state mode A. The 3' cRNA end is coloured in gold and nucleotides are numbered from the 3' to the 5', with C1 and G2 being in the respective -1/+1 active site position. TiLV RdRp motifs are coloured as in (a). Water molecules and magnesium ions (Mg2+) are respectively represented as red and green spheres. Hydrogen bonds are represented as grey dotted lines. Right: as in (a) with nucleotide differences between vRNA and cRNA coloured in red.

c. Left: TiLV polymerase active site in vRNA initiation state. Annotations as in (a) with water molecules and magnesium ions (Mg2+) represented as red and green spheres respectively. The incoming CTP in the +1 active site position is coloured in dark grey. Right: as in (a).

d. Left: TiLV polymerase active site in stalled vRNA elongation state. TiLV RdRp motifs and RNA are coloured as in (a). Water molecules and magnesium ions (Mg2+) are respectively represented as red and green spheres. Hydrogen bonds are represented as grey dotted lines. The incoming CpNHpp in the +1 active site position is coloured in dark grey. For more clarity, only the last incorporated nucleotide from the product is shown, and coloured in cyan. Right: as in (a) with the +1 active site position and the CpNHpp indicated with a dotted rectangle. The 13-mer capped primer ending in ...CC-3' is coloured in magenta and the incorporated nucleotides are coloured in cyan.

SUPPLEMENTARY FIGURE 8

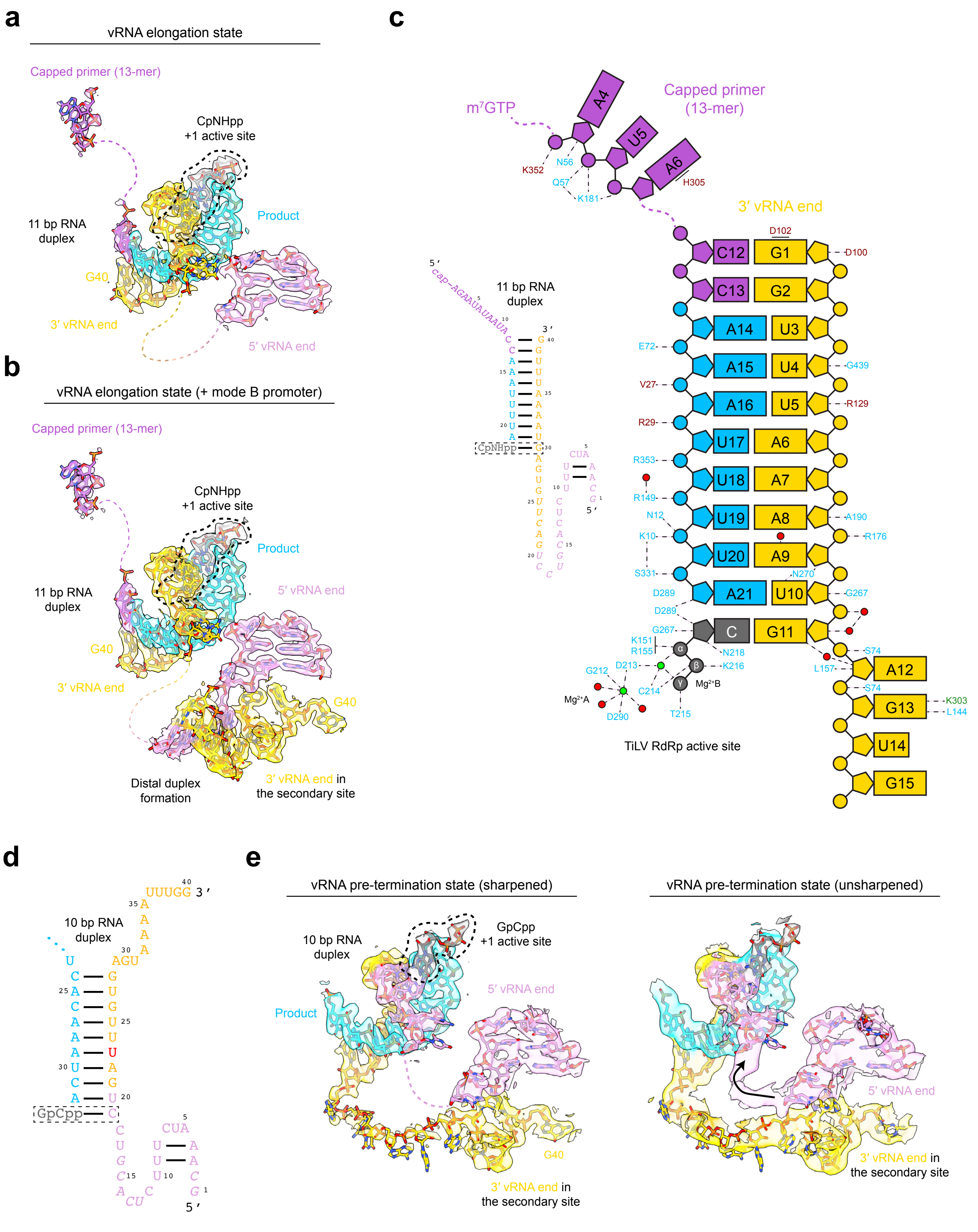

Supplementary Fig. 8. TiLV polymerase in early and late elongation states.

a. RNA density extracted from the cryo-EM map of TiLV polymerase in the elongation state with the v40 template. The 5' vRNA end is in pink, the 3' end in gold. The 13-mer capped primer is in magenta and the incorporated nucleotides in blue. The CpNHpp in the +1 active site position is dark grey. Flexible nucleotides are represented as dotted lines.

b. RNA density extracted from the map of TiLV polymerase in the elongation state with distal duplex. Excess 3' vRNA end is able to bind in the secondary site, reforming a distal duplex with the 5' vRNA end. The RNA colour code is identical to (a).

c. Left: Schematic of the RNA conformation in the early elongation state. CpNHpp is indicated at the +1 position with a dotted rectangle. The capped primer ending by ...CC-3' (13-mer) is in magenta and the incorporated nucleotides are in cyan. TiLV polymerase encloses an 11 bp RNA duplex in this structure. Right: Interactions between TiLV polymerase, the transcription-like product and the 3' vRNA template with RNA colours as in (a). The 3' end nucleotides are numbered from the 3' to the 5'. Flexible nucleotides are dotted. Water molecules and magnesium ions (Mg2+) are respectively represented as red and green spheres. Interacting residues are coloured by their subunit origin, PA (green), PB1 (blue) and PB2-N (dark-red).

d. Schematic of the overall RNA conformation of TiLV polymerase in the pre-termination state with the v40-S template (the mutation “C23U” is highlighted in red). The +1 active site position and the GpCpp are indicated with a dotted rectangle. The only visible incorporated nucleotides of the capped product are in cyan. TiLV polymerase encloses a 10 bp RNA duplex in this structure. Flexible nucleotides are in italic or represented as dotted points.

e. RNA density extracted from the sharpened (left) and unsharpened (right) cryo-EM maps of TiLV polymerase in the pre-termination state, annotated as in (a). The incorporated nucleotides are coloured in cyan. The GpCpp in the +1 active site position is dark grey. The unsharpened map shows continuous template density from the 5' to the 3' end with unbuildable nucleotides indicated by the black arrow.

SUPPLEMENTARY FIGURE 9

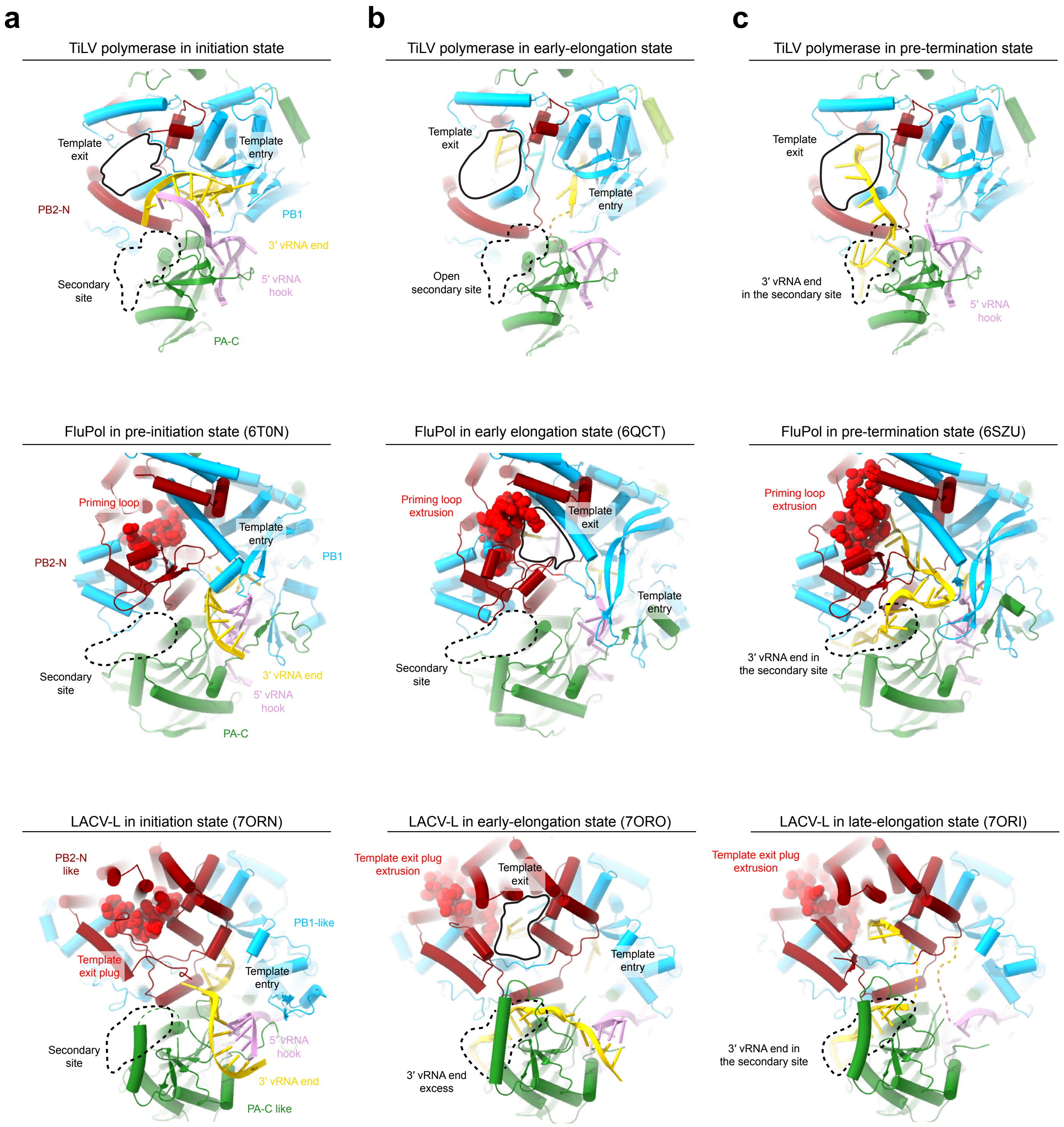

Supplementary Fig. 9. Comparison of TiLV polymerase initiation and elongation states with FluPol and LACV-L.
a. Structural comparison of TiLV polymerase in initiation state with FluPol in pre-initiation state and LACV-L in initiation state. PA is coloured in green, PB1 in light blue and PB2-N in dark red. 5' vRNA ends are coloured in pink. 3' vRNA ends are coloured in gold. FluPol priming loop and LACV-L template exit plug are coloured in red and residues are shown as spheres. The template entry, template exit, and the secondary site are indicated. PDB IDs are indicated.
b. Structural comparison of TiLV polymerase, FluPol and LACV-L in their respective early-elongation states. Domains and RNAs are shown and coloured as in (a). Upon elongation, FluPol priming loop and LACV-L template exit plug are extruded. The template entry, template exit, and the secondary site are indicated. PDB IDs are indicated.
c. Structural comparison of TiLV polymerase and FluPol in in the pre-termination state with LACV-L in the late-elongation state. Domains and RNAs are shown and coloured as in a. Upon late-elongation, the 3' end is able to bind back to the secondary site. Flexible nucleotides are shown as dotted line. The template entry, template exit, and the secondary site are indicated.PDB IDs are indicated.

SUPPLEMENTARY FIGURE 10

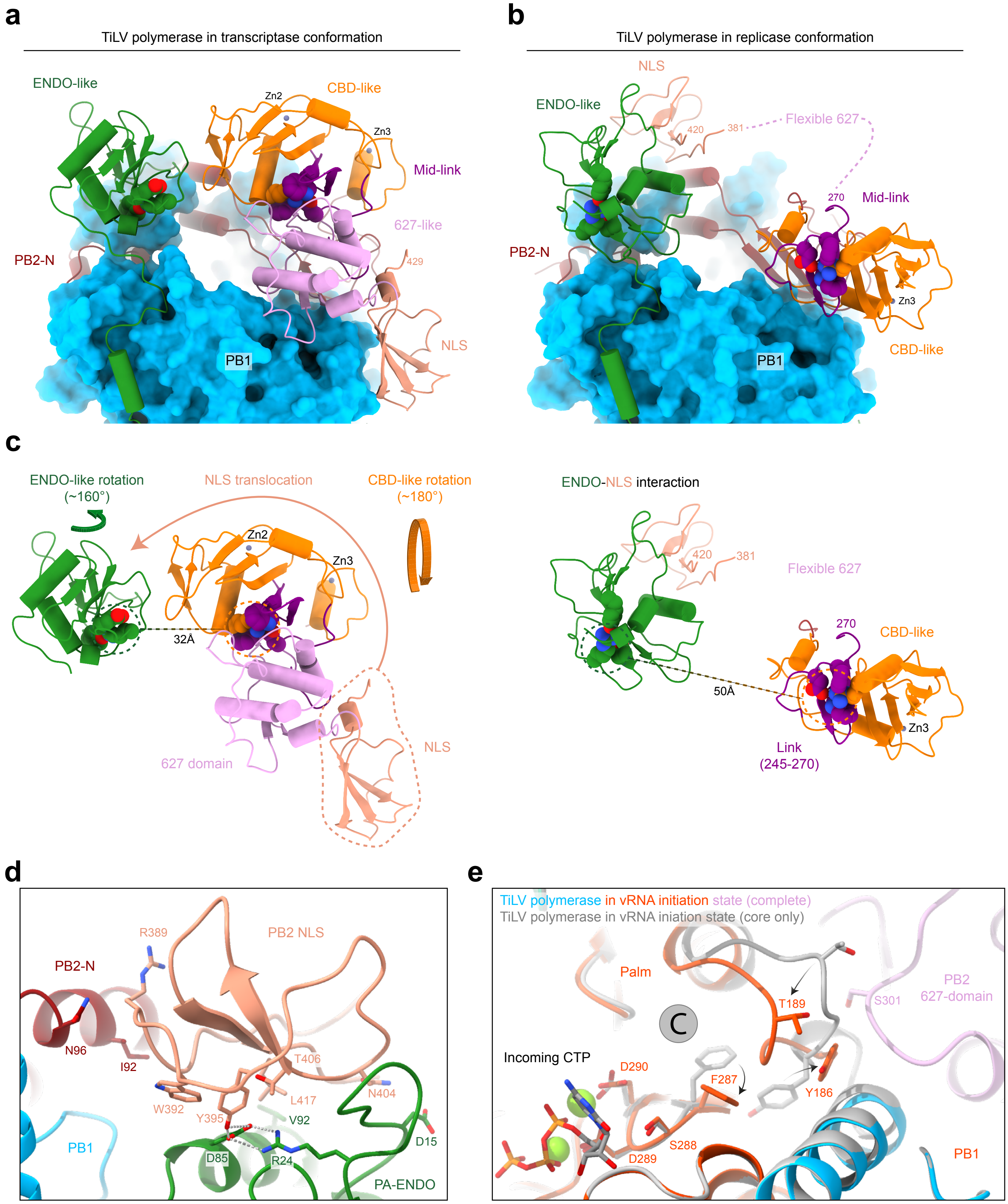

Supplementary Fig. 10. Alternative configurations of TiLV polymerase.
a. TiLV polymerase transcriptase conformation. Close-up view of PA ENDO-like, PB2-N, and PB2-C domains coloured as in Fig. 1d. TiLV polymerase ENDO-like and CBD-like residues analogous to those in the respective FluPol ENDO active site and FluPol cap-binding site are shown as spheres. The PB1 subunit is displayed as a blue surface.
b. TiLV polymerase replicase conformation, annotated as in (a). TiLV PB2 627 domain is flexible in this conformation.
c. The transcriptase to replicase transition. Domains are coloured as in (a). To switch from transcriptase to replicase, PA ENDO-like domain rotates ~160 degrees and PB2 NLS domain translocates to interact with it. PB2 CBD-like domain rotates ~180 degrees and packs against TiLV polymerase PB1 subunit (left). In the transcriptase conformation, TiLV polymerase PA ENDO-like and PB2 CBD-like residues, analogous to the respective FluPol PA ENDO active site and FluPol PB2 cap-binding site, are 32 Å apart. In the replicase conformation the corresponding residues are 50 Å apart (right).
d. Close-up view of the TiLV polymerase PA ENDO-like / PB2 NLS domain interaction. Domains are coloured as in (a). Hydrogen bonds are shown as grey dotted lines. Key interacting residues are shown as sticks.
e. In full-length structures, compared to core-only structures, packing of TiLV polymerase 627-like domain against the PB1 subunit induces a conformational change that propagates towards the RdRp active site. The PB2 627-like domain is coloured in pink. PB1 subunit is coloured in light blue with the palm domain in orange. The RdRp motif C (285-295) is indicated. The main movements between TiLV polymerase in vRNA initiation state (core only and complete structure) are indicated with arrows. TiLV polymerase structures are aligned with each other using PB1 as reference.

Supplementary Table 1a. Cryo-EM data collection, refinement and validation statistics of TiLV polymerase structures (samples 1-3).

	vRNA initiation		cRNA pre-initiation mode A		cRNA pre-initiation mode B	vRNA pre-initiation mode A
**Structure No.**	1	2	3	4	5	6
**Short name**	Full vRNA initiation with CTP	Core vRNA initiation with CTP	Core cRNA pre-initiation mode A	Core with endo cRNA pre-initiation mode A	Closed core with endo cRNA mode B	Core vRNA pre-initiation mode A
	PDB ID 8PSN, EMD-17857	PDB ID 8PSO, EMD-17858	PDB ID 8PSQ, EMD-17860	PDB ID 8PT7, EMD-17869	PDB ID 8PSS, EMD-17861	PDB ID 8PSU, EMD-17862
**Data collection and processing**	ESRF CM01 ThermoFisher Krios TEM | Gatan K3 direct electron detector mounted on a Gatan Bioquantum LS/967 energy filter		ESRF CM01 ThermoFisher Krios TEM | Gatan K3 direct electron detector mounted on a Gatan Bioquantum LS/967 energy filter			ThermoFisher Glacios TEM | Gatan K2 Summit
**Magnification**	105000		105000			36000
**Voltage (kV)**	300		300			200
**Electron exposure (e–/Å²)**	51		51			40
**Defocus range (µm)**	-0.8 / -2.0		-0.8 / -2.0			-0.8 / -2.0
**Pixel size (Å)**	0.84		0.84			1.1
**Symmetry imposed**	C1		C1			C1
**Initial/Final micrographs**	6000 / 4133		6000 / 3463			968 / 953
**Final particle images (no.)**	46595	271170	107798	153101	109188	67042
**Map resolution (Å) FSC threshold 0.143**	2.73	2.40	2.65	2.80	2.83	3.18
**Map resolution range (Å)**	2.6-3.8	2.3-3.1	2.6-3.8	2.6-3.8	2.6-3.8	3.2-4.0
**Refinement**						
**Model resolution (Å) FSC threshold 0.5**	2.7	2.4	2.6	2.8	2.8	3.2
**Map sharpening *B* factor (Å²)**	-40	-54	-58	-40	-40	-81
**CC (mask)**	0.8726	0.8841	0.8868	0.8949	0.8870	0.8198
**Composition**<br>**Non-H atoms**<br>**Protein**<br>**Nucleotides**<br>**Water**<br>**Ligands**	 11301<br>1361<br>29<br>2<br>3 x Zn 2 x Mg	 8264<br>971<br>29<br>44<br>1 x Zn 2 x Mg	 8181<br>969<br>28<br>1<br>1 x Zn 1 x Mg	 9032<br>1071<br>29<br>0<br>1 x Zn 1 x Mg	 8697<br>1068<br>15<br>0<br>1 x Zn 1 x Mg	 8212<br>971<br>29<br>0<br>1 x Zn
***B* factors (Å²)**<br>**Protein**<br>**Nucleotides**<br>**Water**<br>**Ligands**	 38.03<br>63.64<br>19.39<br>66.15	 32.03<br>54.21<br>24.31<br>38.48	 36.52<br>65.87<br>72.78<br>23.03	 66.89<br>102.87<br>-<br>62.76	 72.72<br>85.07<br>-<br>65.56	 49.64<br>91.53<br>-<br>72.80
**RMS deviations**<br>**Bond lengths (Å)**<br>**Bond angles (°)**	 0.002<br>0.519	 0.002<br>0.482	 0.003<br>0.471	 0.002<br>0.436	 0.002<br>0.440	 0.003<br>0.446
**Validation**<br>**MolProbity score**<br>**Clashscore**<br>**Poor rotamers (%)**	 1.50<br>3.50<br>2.57	 1.18<br>2.41<br>1.55	 1.14<br>2.79<br>1.19	 1.35<br>3.66<br>1.29	 1.55<br>2.96<br>1.73	 1.22<br>3.46<br>0.0
**Ramachandran**<br>**Favored (%)**<br>**Allowed (%)**<br>**Disallowed (%)**	 97.79<br>2.14<br>0.07	 97.93<br>2.07<br>0.0	 97.92<br>1.97<br>0.10	 97.46<br>2.44<br>0.09	 95.85<br>4.15<br>0.0	 97.62<br>2.18<br>0.21

Supplementary data Table 1b. Cryo-EM data collection, refinement and validation statistics of TiLV polymerase structures (sample 4)

	**vRNA elongation**		**vRNA mode B**			**Replicase**
**Structure No.**	7	8	9	10	11	12
**Short name**	Full vRNA elongation with CpNHpp	Full vRNA elongation with CpNHpp and additional mode B promoter	Full with open core vRNA mode B	Open core with rotated endo-NLS vRNA mode B	Closed core with rotated endo-NLS vRNA mode B	Replicase initiation with CpNHpp
	PDB ID 8PSX, EMD-17864	PDB ID 8PSZ, EMD-17865	PDB ID 8PT2, EMD-17866	PDB ID 8PTH, EMD-17871	PDB ID 8PTJ, EMD-17872	PDB ID 8PT6, EMD-17868
**Data collection and processing**	ESRF CM01 ThermoFisher Krios TEM | Gatan K3 direct electron detector mounted on a Gatan Bioquantum LS/967 energy filter					
**Magnification**	105000					
**Voltage (kV)**	300					
**Electron exposure (e–/Å^2)**	51					
**Defocus range (µm)**	-0.8 / -2.0					
**Pixel size (Å)**	0.84					
**Symmetry imposed**	C1					
**Final particle images (no.)**	29892	103012	80382	36587	53296	80175
**Initial/Final micrographs (no.)**	6000 / 5831					
**Map resolution (Å)<br>FSC threshold 0.143**	2.96	2.42	2.59	2.73	2.86	2.9
**Map resolution range (Å)**	2.6-4.2	2.3-3.5	2.4-4.0	2.5-4.1	2.5-4.1	2.8-4.8
**Refinement**						
**Model resolution (Å)<br>FSC threshold 0.5**	2.9	2.4	2.6	2.7	2.9	3.0
**Map sharpening *B* factor (Å^2)**	-40	-30	-20	-20	-20	-60
**CC (mask)**	0.8368	0.8803	0.8835	0.8854	0.8938	0.8378
**Composition<br>Non-H atoms<br>Protein residues<br>Nucleotides<br>Water<br>Ligands**	 11486<br>1357<br>38<br>0<br>3 x Zn 2 x Mg	 11903<br>1356<br>53<br>109<br>3 x Zn 2 x Mg	 11190<br>1355<br>27<br>1<br>3 x Zn	 9245<br>1104<br>27<br>1<br>1 x Zn	 9034<br>1102<br>15<br>2<br>1 x Zn	 10397<br>1243<br>29<br>0<br>3 x Zn 2 x Mg
***B* factors (Å^2)<br>Protein<br>Nucleotides<br>Water<br>Ligand**	 37.62<br>39.86<br>-<br>81.58	 29.60<br>44.81<br>16.22<br>60.93	 54.98<br>98.50<br>28.66<br>115.45	 59.26<br>82.32<br>36.24<br>91.94	 65.11<br>72.32<br>44.34<br>101.42	 80.08<br>95.52<br>-<br>122.29
**R.m.s. deviations<br>Bond lengths (Å)<br>Bond angles (°)**	 0.003<br>0.505	 0.009<br>0.866	 0.002<br>0.446	 0.002<br>0.438	 0.004<br>0.485	 0.003<br>0.535
**Validation<br>MolProbity score<br>Clashscore<br>Poor rotamers (%)**	 1.61<br>3.37<br>1.71	 1.57<br>2.52<br>2.06	 1.36<br>2.40<br>1.37	 1.64<br>3.02<br>1.88	 1.80<br>3.52<br>2.82	 1.64<br>5.67<br>1.59
**Ramachandran<br>Favored (%)<br>Allowed (%)<br>Disallowed (%)**	 95.69<br>4.31<br>0.00	 95.69<br>4.31<br>0.00	 96.43<br>3.57<br>0.0	 95.16<br>4.84<br>0.0	 95.50<br>4.50<br>0.0	 96.92<br>2.92<br>0.16

Supplementary Table 1c. Cryo-EM data collection, refinement and validation statistics of TiLV polymerase structures (sample 5)

	**Pre-termination**
**Structure No.**	13
**Short name**	Full vRNA pre-termination with GpCpp
	PDB 8QZ8<br>EMD-18772
**Data collection and processing**	ThermoFisher Glacios TEM | F4i - SelectrisX
**Magnification**	130000
**Voltage (kV)**	200
**Electron exposure (e–/Å^2)**	40
**Defocus range (µm)**	-0.8 / -2.0
**Pixel size (Å)**	0.878
**Symmetry imposed**	C1
**Final particle images (no.)**	107403
**Initial/Final micrographs (no.)**	2433 / 2253
**Map resolution (Å) FSC threshold 0.143**	3.13
**Map resolution range (Å)**	3.1-4.0
**Refinement**	
**Model resolution (Å) FSC threshold 0.5**	3.3
**Map sharpening *B* factor (Å^2)**	-116
**CC (mask)**	0.8385
**Composition**<br>**Non-H atoms**<br>**Protein residues**<br>**Nucleotides**<br>**Water**<br>**Ligands**	 11552<br>1356<br>42<br>0<br>3 x Zn 1 x Mg
***B* factors (Å^2)**<br>**Protein**<br>**Nucleotides**<br>**Water**<br>**Ligand**	 81.90<br>96.46<br>-<br>106.25
**R.m.s. deviations**<br>**Bond lengths (Å)**<br>**Bond angles (°)**	 0.003<br>0.457
**Validation**<br>**MolProbity score**<br>**Clashscore**<br>**Poor rotamers (%)**	 1.38<br>3.62<br>0.09
**Ramachandran**<br>**Favored (%)**<br>**Allowed (%)**<br>**Disallowed (%)**	 96.51<br>3.34<br>0.15

SUPPLEMENTARY NOTE 1

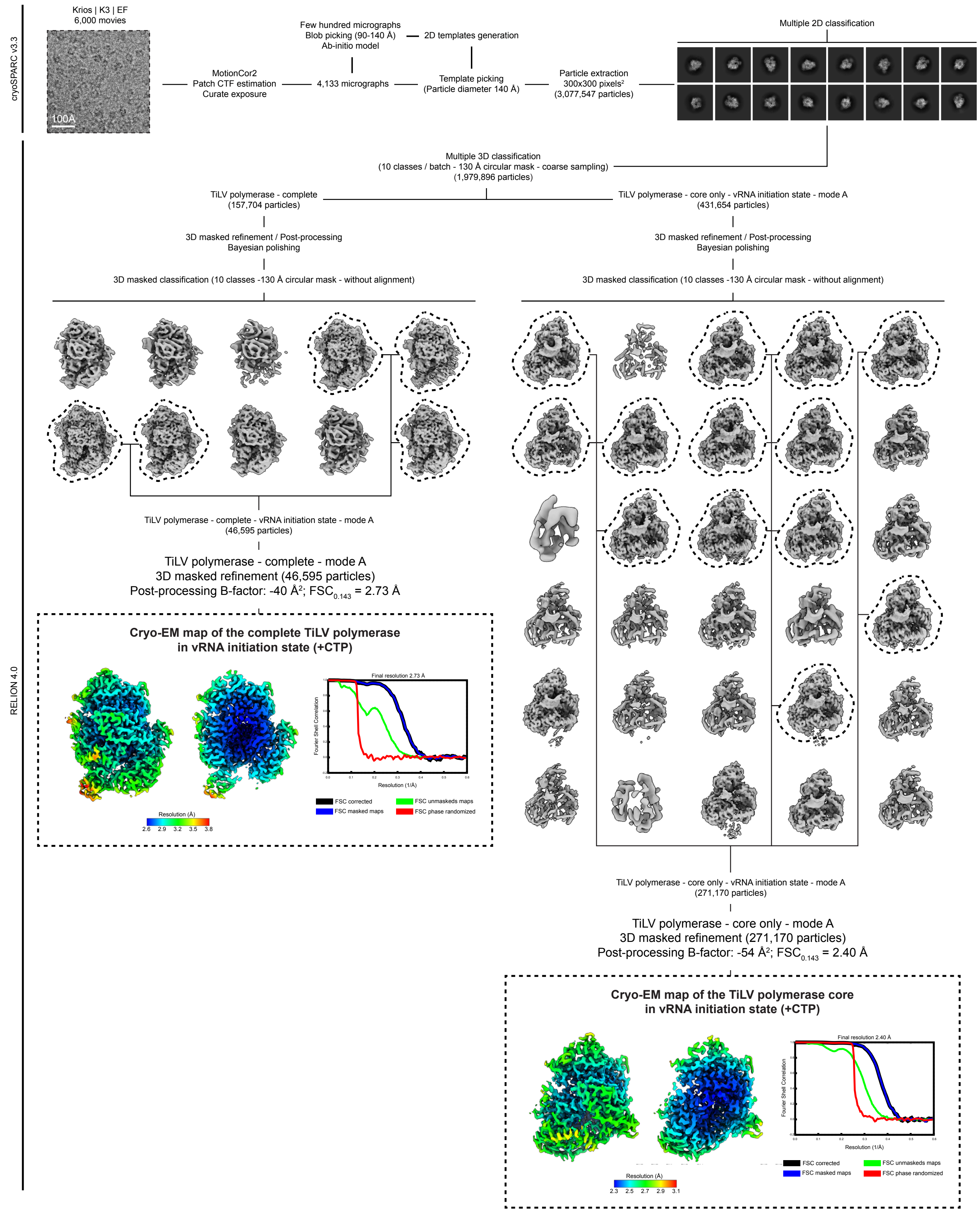

Supplementary Note 1. Cryo-EM image processing strategy applied to obtain TiLV polymerase structures in the vRNA initiation state.
Schematics of the image processing strategy used with the data collected on a Titan Krios equipped with a Gatan K3 direct electron detector mounted on a Gatan Bioquantum energy filter. Representative cropped micrograph, 2D class averages and 3D class averages are displayed. Full and cutaway views of each local resolution filtered EM maps are shown. Fourier shell correlation curves (FSC) are displayed. Scale bar = 100 Å.

SUPPLEMENTARY NOTE 2

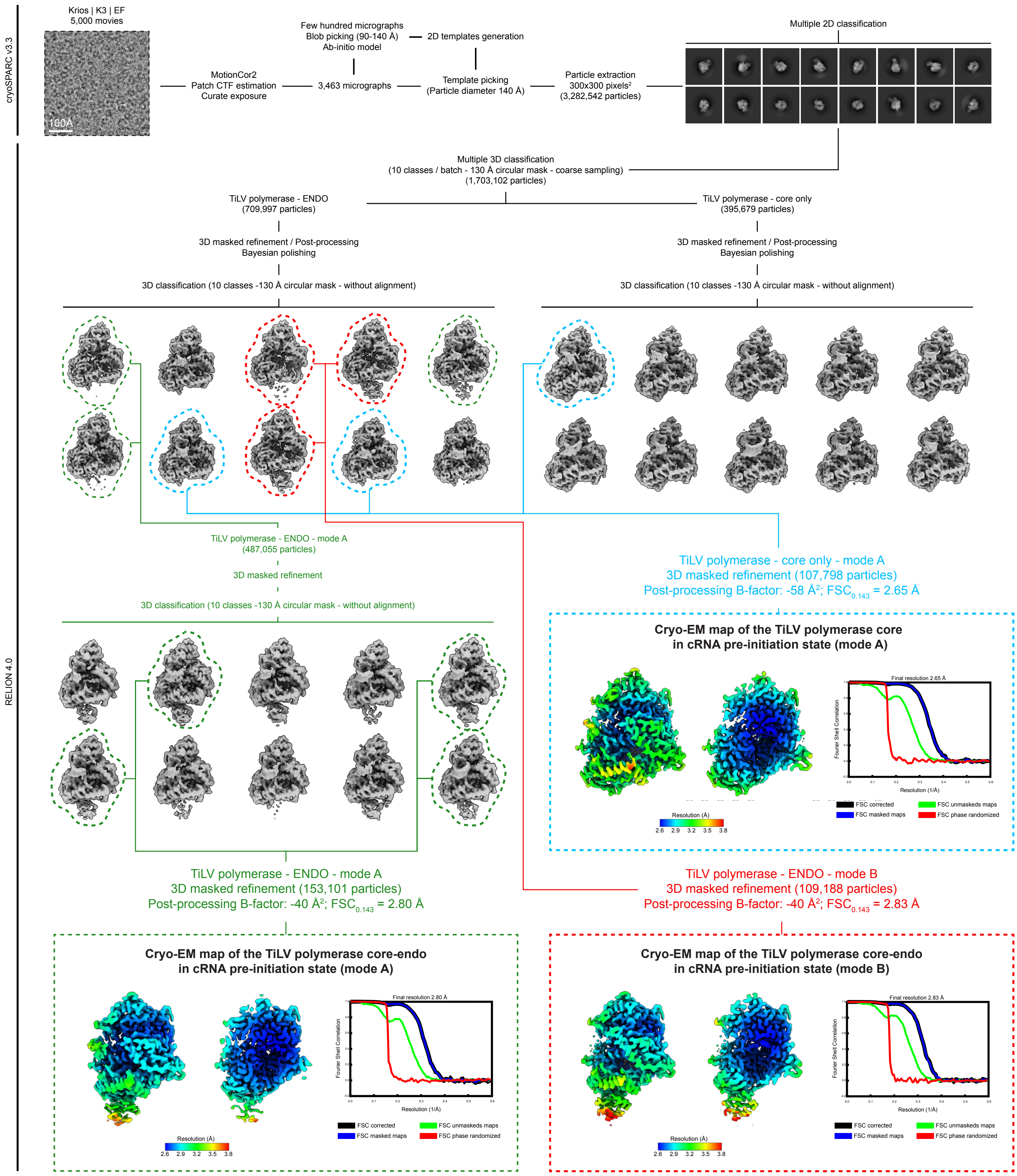

Supplementary Note 2. Cryo-EM image processing strategy applied to obtain TiLV polymerase structures in the cRNA pre-initiation state (mode A/B). Schematics of the image processing strategy used with the data collected on a Titan Krios equipped with a Gatan K3 direct electron detector mounted on a Gatan Bioquantum energy filter. Representative cropped micrograph, 2D class averages and 3D class averages are displayed. Full and cutaway views of each local resolution filtered EM maps are shown. Fourier shell correlation curves (FSC) are displayed. Scale bar = 100 Å.

SUPPLEMENTARY NOTE 3

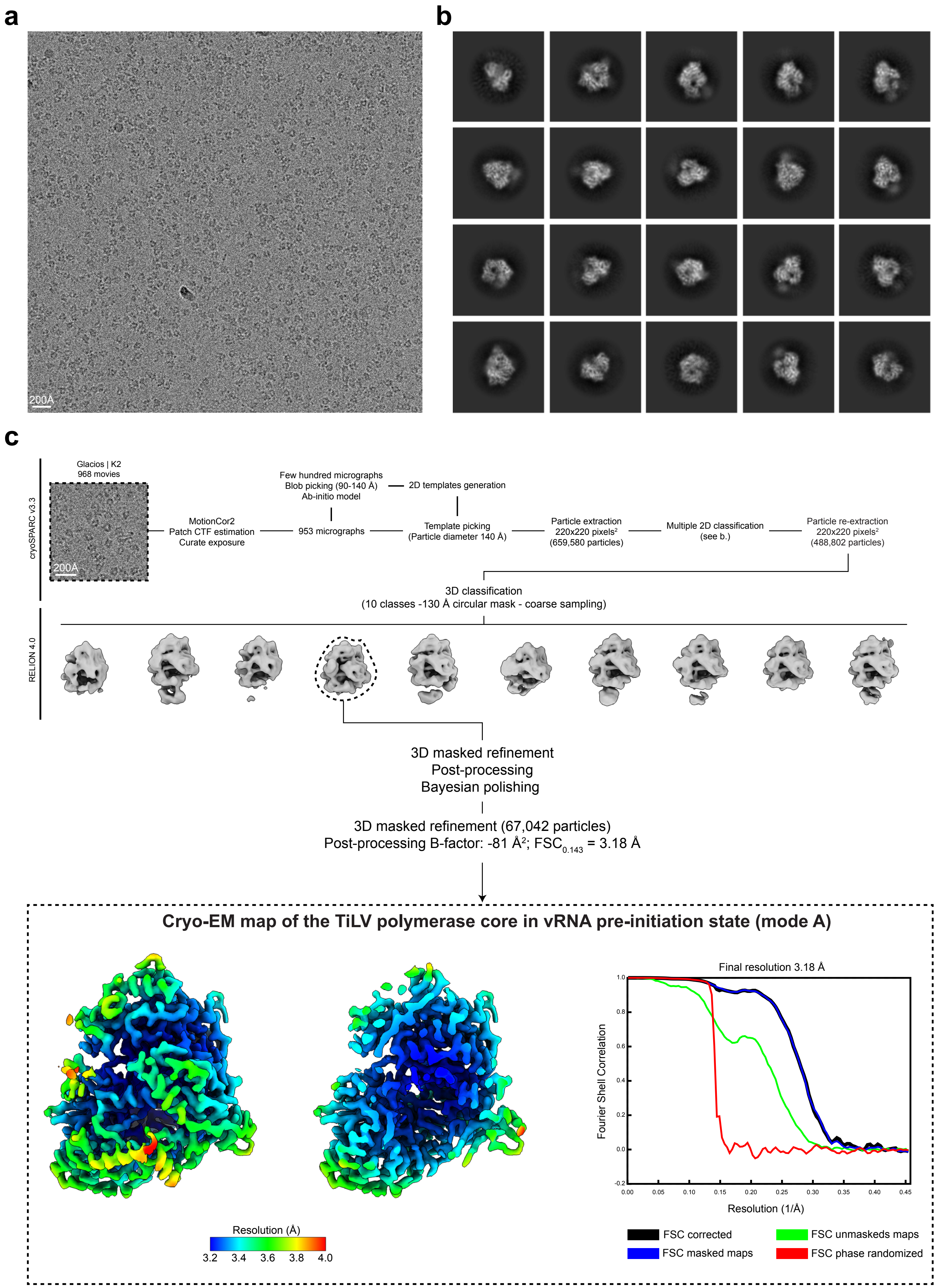

Supplementary Note 3. Cryo-EM image processing strategy applied to obtain TiLV polymerase structure in vRNA pre-initiation state (mode A).
a. Representative realigned micrograph. Scale bar = 200 Å.
b. Representative 2D class averages obtained from cryoSPARC.
c. Schematics of the image processing strategy used with the data collected on a Glacios equipped with a Gatan K2 direct electron detector. Representative cropped micrograph (see a) and 3D class averages are displayed. Full and cutaway views of the local resolution filtered EM map are shown. Fourier shell correlation curves (FSC) are displayed.

SUPPLEMENTARY NOTE 4

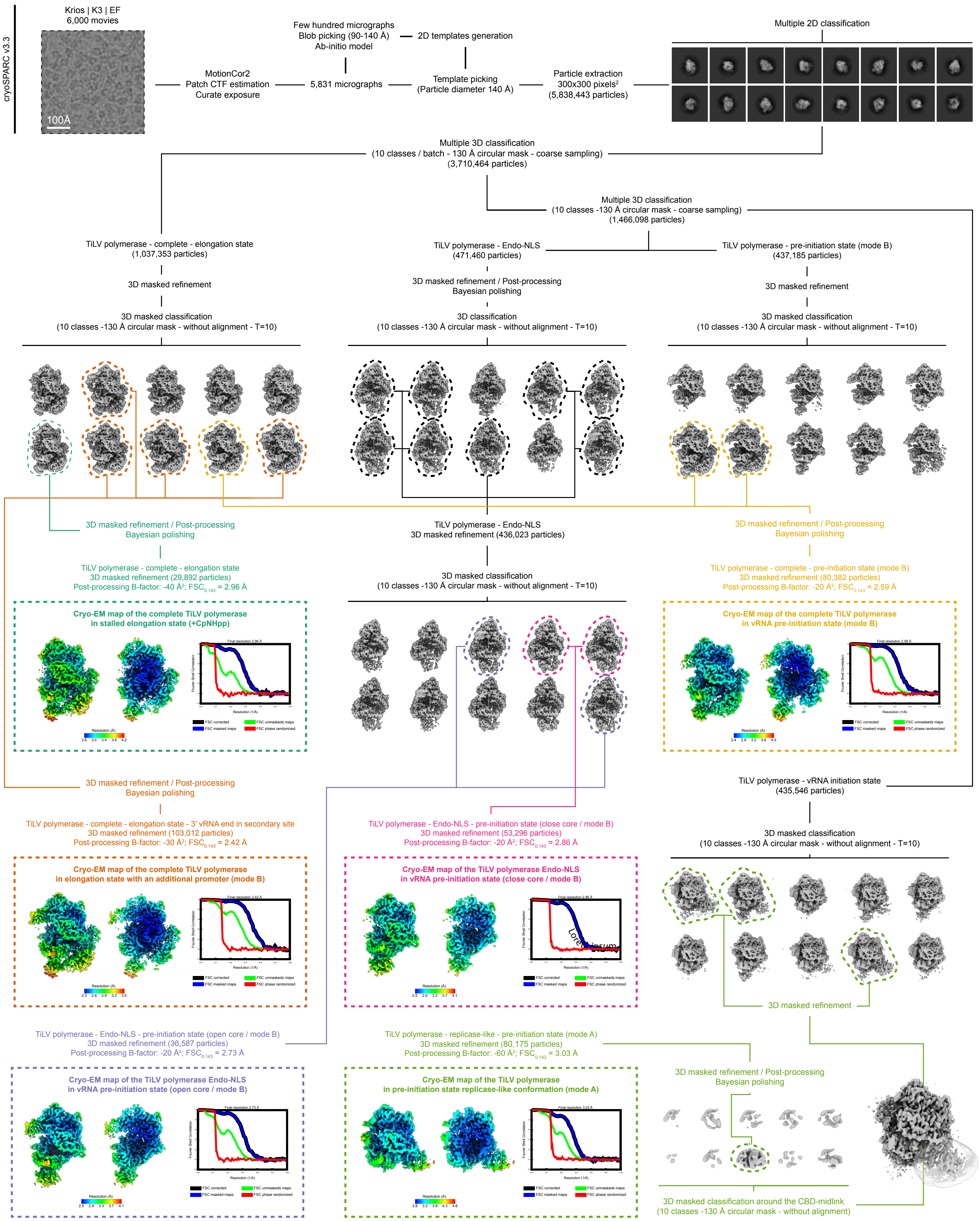

Supplementary Note 4. Cryo-EM image processing strategy applied to obtain TiLV polymerase structures in the vRNA elongation states, vRNA pre-initiation state (complete mode B, open/close core mode B), and TiLV replicase in vRNA initiation state.
Schematics of the image processing strategy used with the data collected on a Titan Krios equipped with a Gatan K3 direct electron detector mounted on a Gatan Bioquantum energy filter. Representative cropped micrograph, 2D class averages and 3D class averages are displayed. Full and cutaway views of each local resolution filtered EM maps are shown. Fourier shell correlation curves (FSC) are displayed. Scale bar = 100 Å.

SUPPLEMENTARY NOTE 5

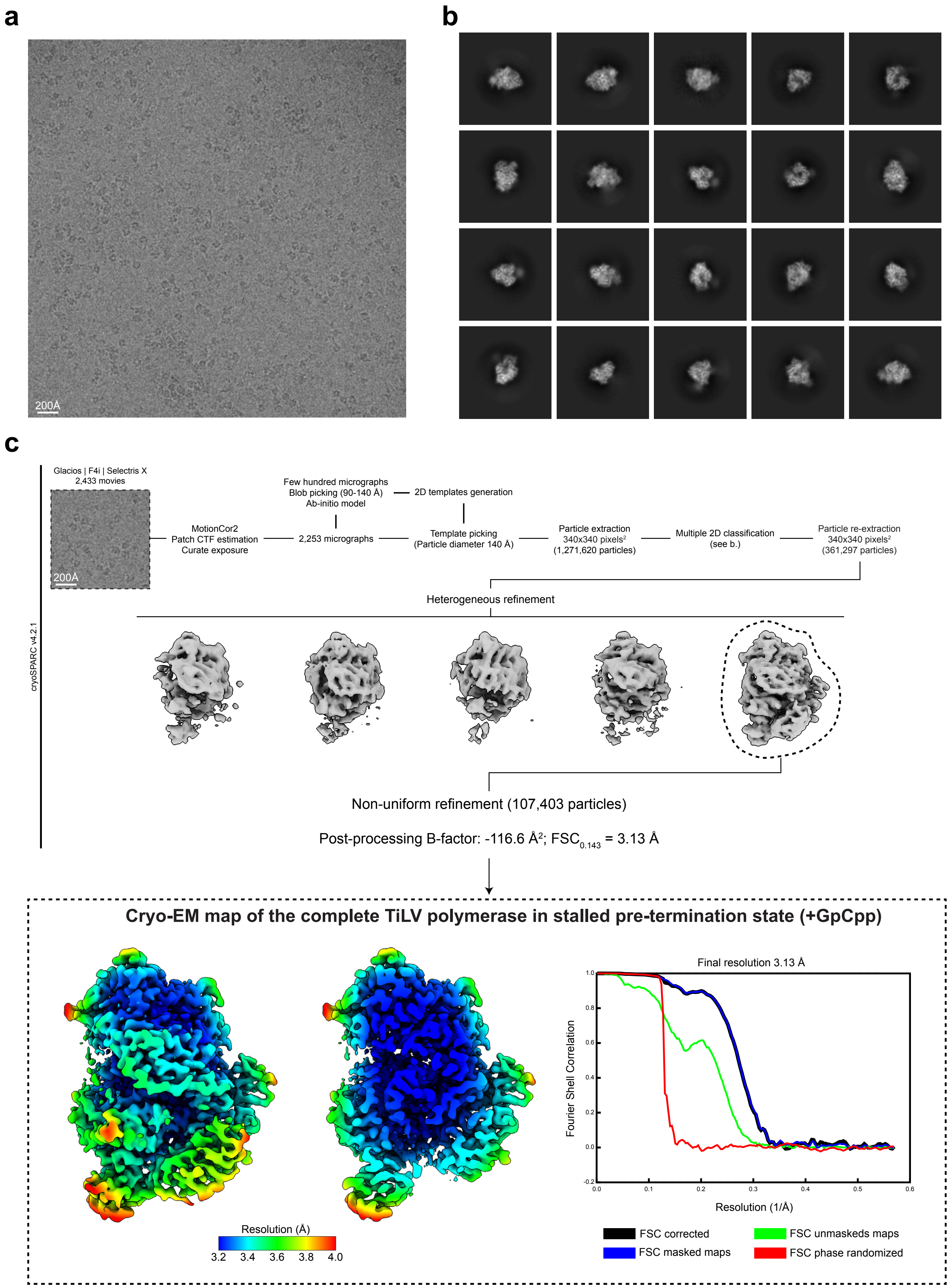

Supplementary Note 5. Cryo-EM image processing strategy applied to obtain the structure of TiLV polymerase in the vRNA pre-termination state.
a. Representative realigned micrograph. Scale bar = 200 Å.
b. Representative 2D class averages obtained from cryoSPARC.
c. Schematics of the image processing strategy used with the data collected on a Glacios equipped with a F4i direct electron detector and a SelectrisX energy filter. Representative cropped micrograph (see a) and 3D class averages are displayed. Full and cutaway views of the local resolution filtered EM map are shown. Fourier shell correlation curves (FSC) are displayed.

SUPPLEMENTARY NOTE 6 (1/2)

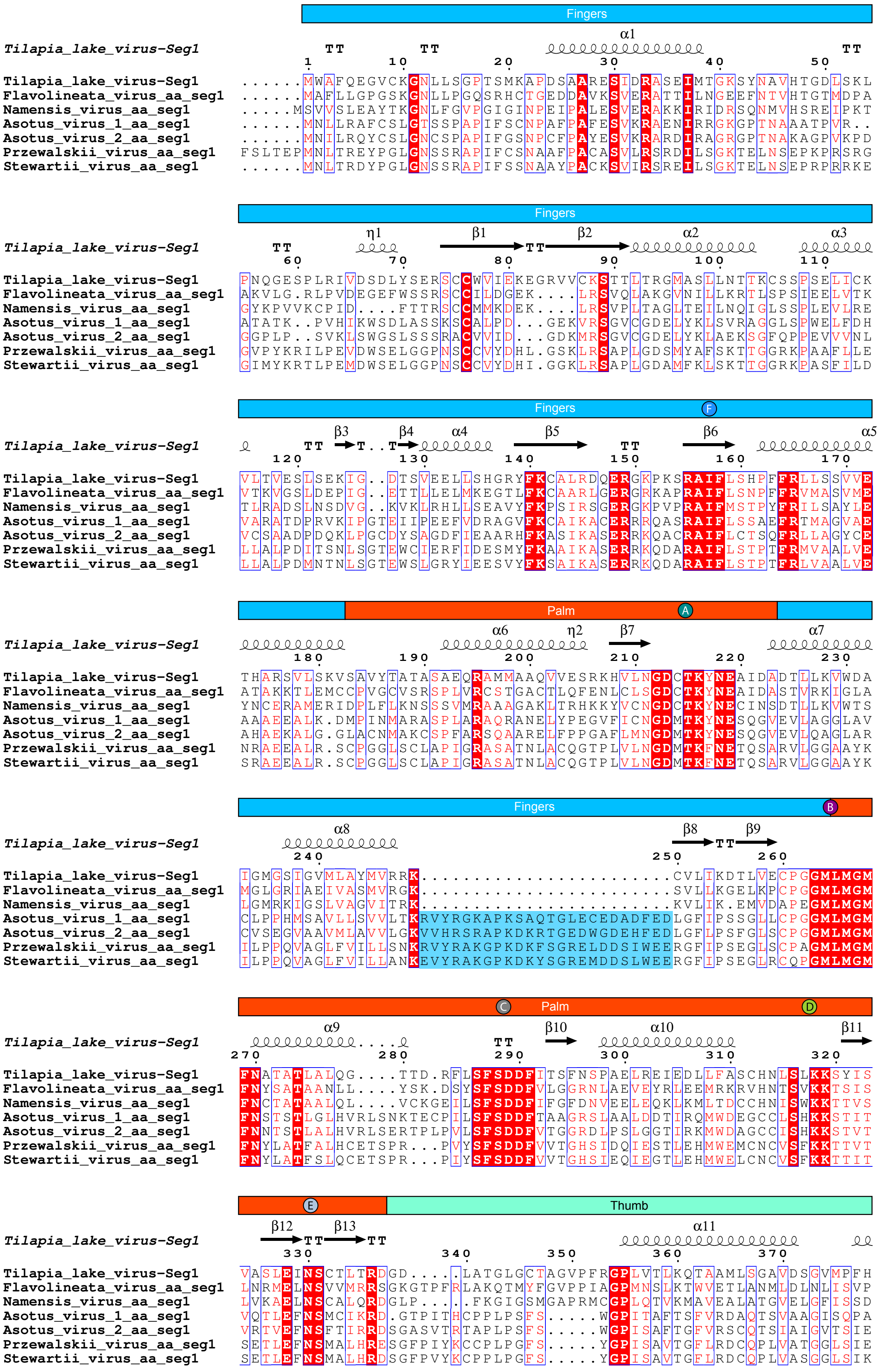

SUPPLEMENTARY NOTE 6 (2/2)

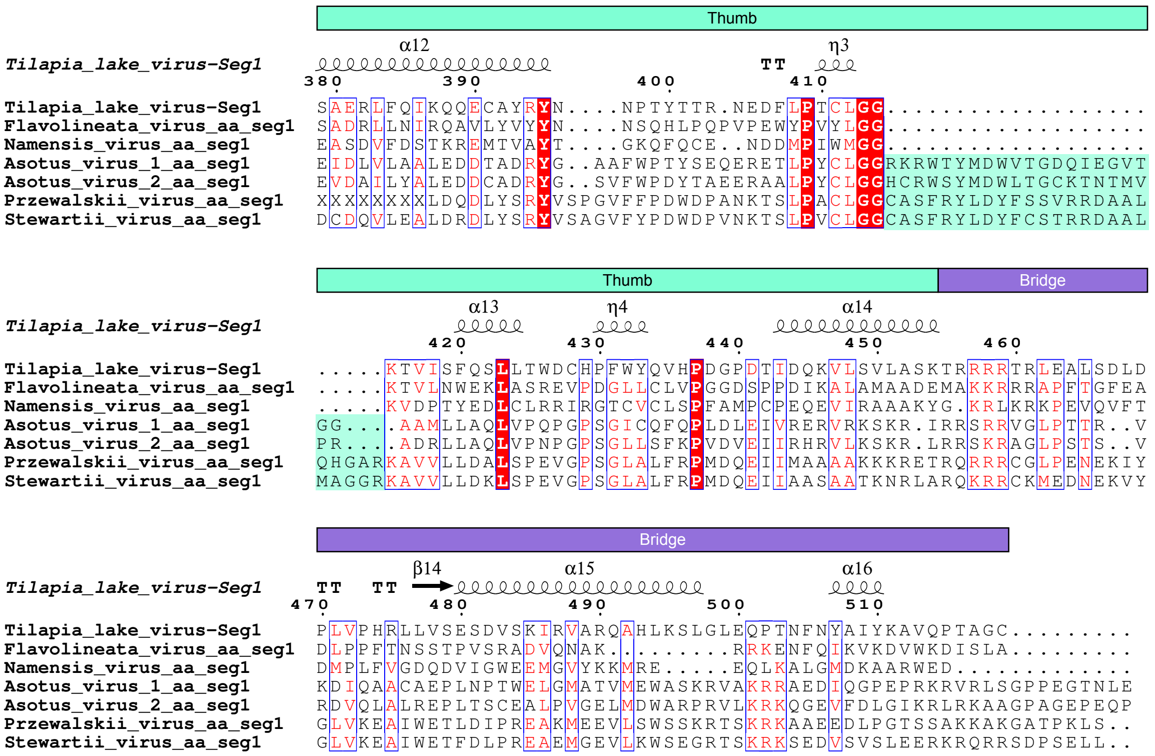

Supplementary Note 6. Multiple alignment of TiLV polymerase PB1 subunit (segment 1) with the putative PB1 from other Amnoonviridae family viruses. TiLV PB1 secondary structures are shown and numbered. Domain positions and TiLV RdRp motifs are indicated. Insertions in the PB2 of Asotus, Przewalskii and Stewartii viruses are indicated with a coloured rectangle.

SUPPLEMENTARY NOTE 7

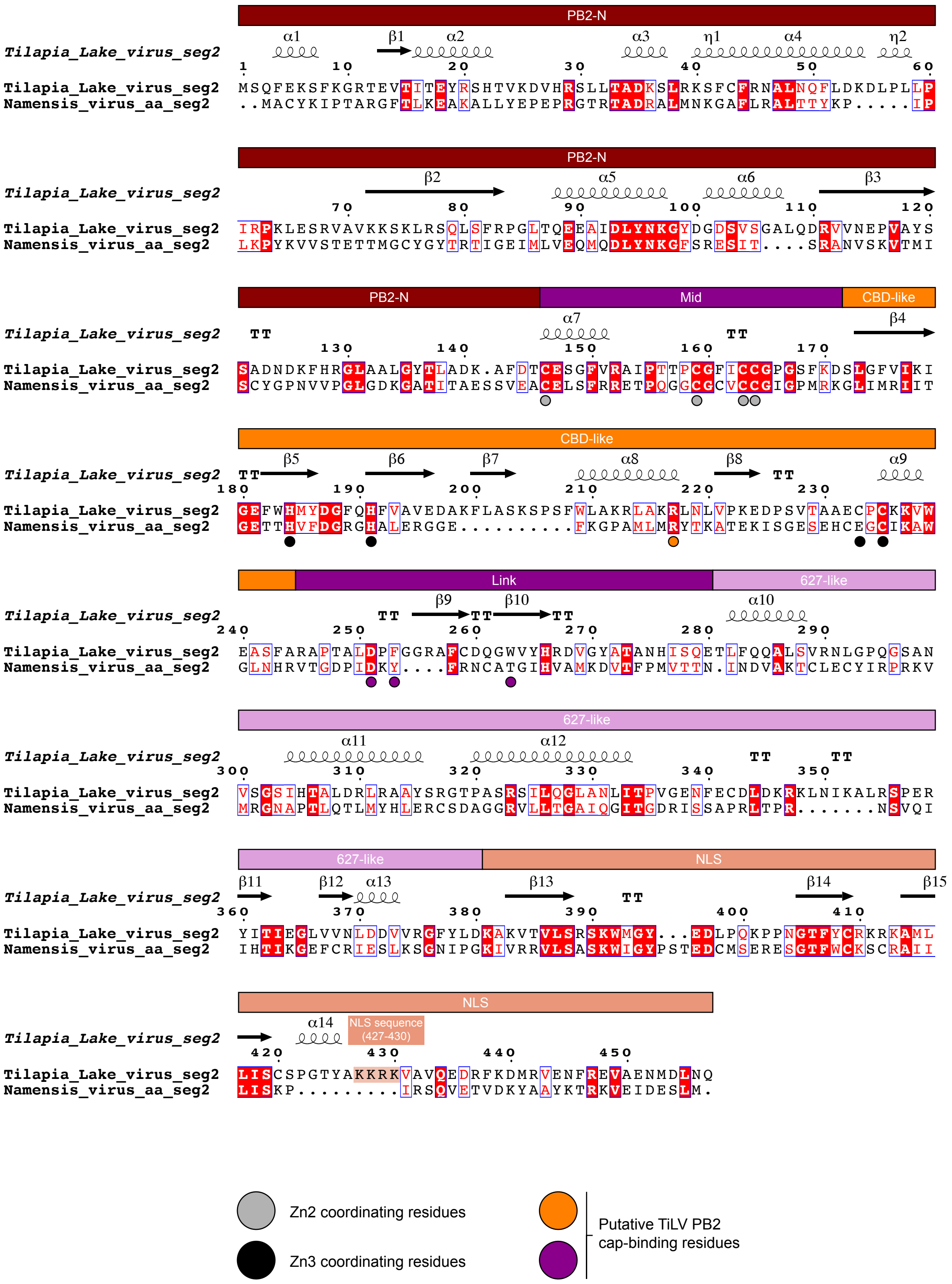

Supplementary Note 7. Multiple alignment of TiLV polymerase PB2 subunit (segment 2) with Namensis virus (segment 2) from the Amnoonviridae family. TiLV PB2 secondary structures are shown and numbered. Domain positions are indicated. Residues coordinating Zn2 and Zn3 are respectively labelled with grey and dark circles. Putative TiLV PB2 cap-binding residues are labelled with orange (PB2 CBD-like) and purple (PB2 link) circles. TiLV NLS sequence (427-430) is indicated in a rectangle.

SUPPLEMENTARY NOTE 8

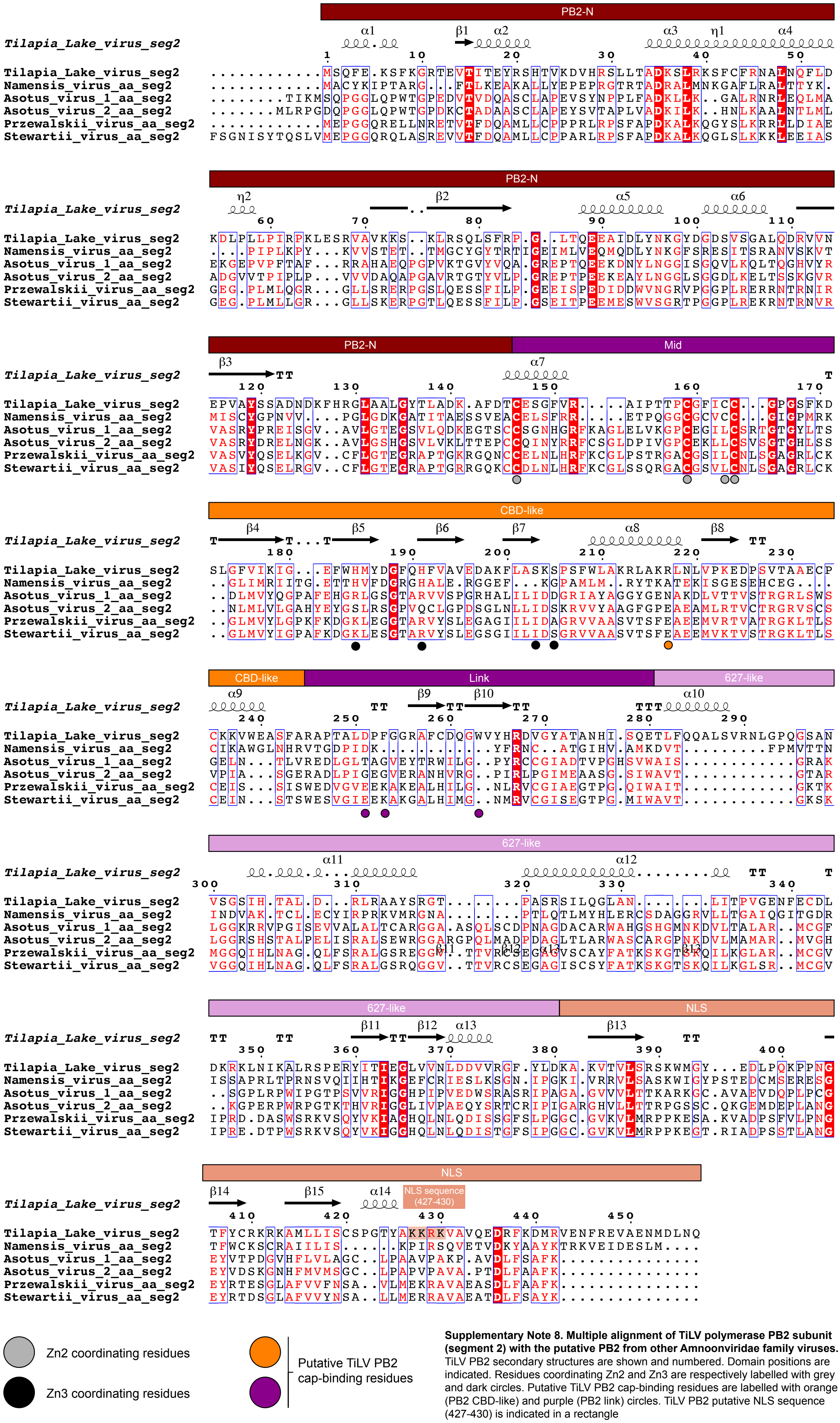

Supplementary Note 8. Multiple alignment of TiLV polymerase PB2 subunit (segment 2) with the putative PB2 from other Amnoonviridae family viruses. TiLV PB2 secondary structures are shown and numbered. Domain positions are indicated. Residues coordinating Zn2 and Zn3 are respectively labelled with grey and dark circles. Putative TiLV PB2 cap-binding residues are labelled with orange (PB2 CBD-like) and purple (PB2 link) circles. TiLV PB2 putative NLS sequence (427-430) is indicated in a rectangle

SUPPLEMENTARY NOTE 9

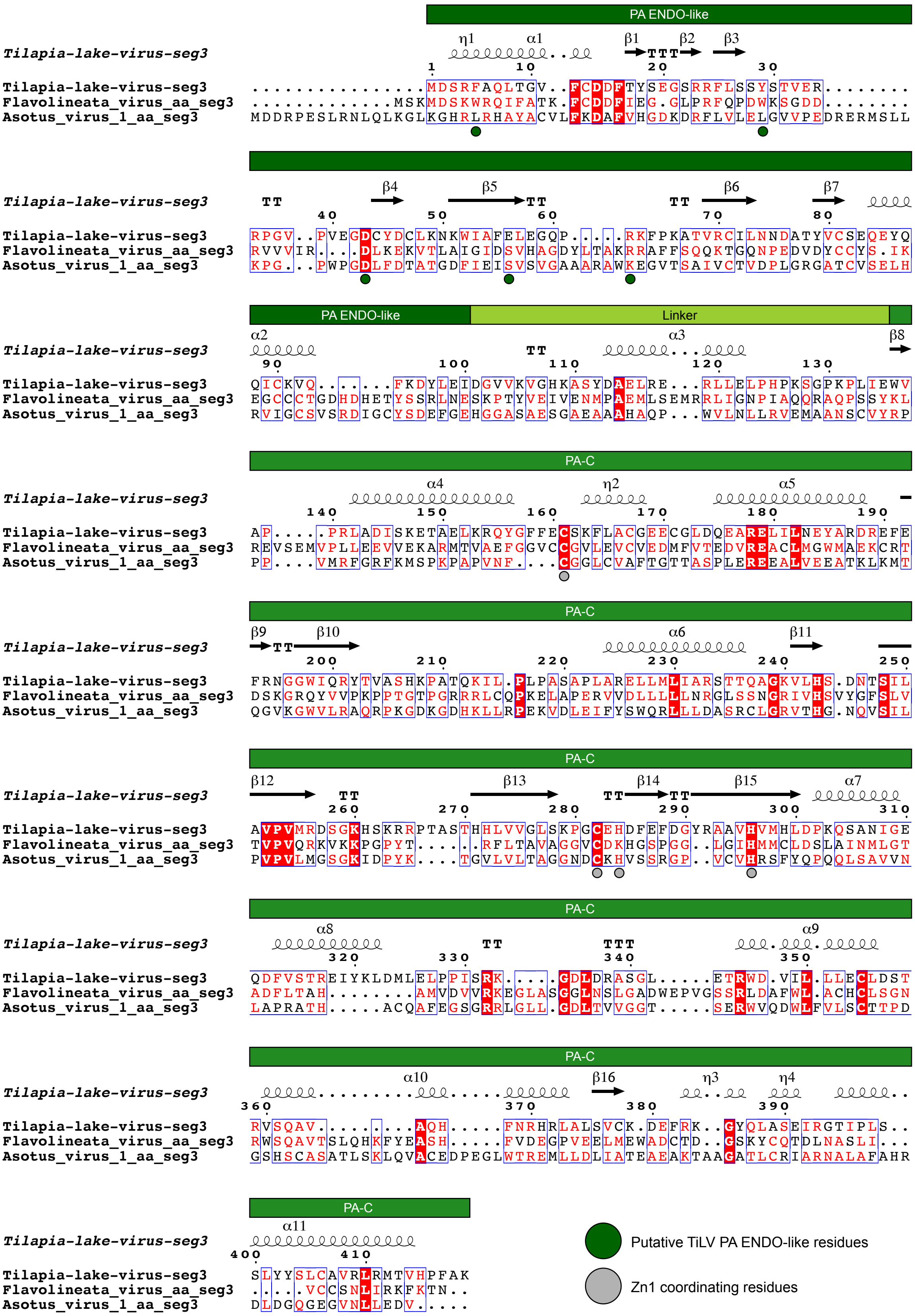

Supplementary Note 9. Multiple alignment of TiLV polymerase PA subunit (segment 3) with the putative PA from other Amnoonviridae family viruses. TiLV PA secondary structures are shown and numbered. Domain positions are indicated. Putative TiLV PA ENDO-like residues are labelled with green circles. Residues coordinating Zn1 are labelled with grey circles.
