# Supplementary material for: Structural and functional analysis of the minimal orthomyxovirus-like polymerase of Tilapia Lake Virus from the highly diverged Amnoonviridae family
Source: Nat Commun. 2023 Dec 9;14:8145. doi: 10.1038/s41467-023-44044-x (PMC10709604; doi:10.1038/s41467-023-44044-x)
Supplement: Supplementary file 3 — Reporting Summary [file 41467_2023_44044_MOESM3_ESM.pdf]

nature portfolio

Corresponding author(s): Stephen Cusack

Last updated by author(s): Nov 20, 2023

Reporting Summary

Nature Portfolio wishes to improve the reproducibility of the work that we publish. This form provides structure for consistency and transparency in reporting. For further information on Nature Portfolio policies, see our Editorial Policies and the Editorial Policy Checklist.

Statistics

For all statistical analyses, confirm that the following items are present in the figure legend, table legend, main text, or Methods section.

n/a	Confirmed	
x		The exact sample size (*n*) for each experimental group/condition, given as a discrete number and unit of measurement
x		A statement on whether measurements were taken from distinct samples or whether the same sample was measured repeatedly
x		The statistical test(s) used AND whether they are one- or two-sided<br>*Only common tests should be described solely by name; describe more complex techniques in the Methods section.*
x		A description of all covariates tested
x		A description of any assumptions or corrections, such as tests of normality and adjustment for multiple comparisons
	x	A full description of the statistical parameters including central tendency (e.g. means) or other basic estimates (e.g. regression coefficient) AND variation (e.g. standard deviation) or associated estimates of uncertainty (e.g. confidence intervals)
x		For null hypothesis testing, the test statistic (e.g. *F*, *t*, *r*) with confidence intervals, effect sizes, degrees of freedom and *P* value noted<br>*Give P values as exact values whenever suitable.*
x		For Bayesian analysis, information on the choice of priors and Markov chain Monte Carlo settings
x		For hierarchical and complex designs, identification of the appropriate level for tests and full reporting of outcomes
x		Estimates of effect sizes (e.g. Cohen's *d*, Pearson's *r*), indicating how they were calculated

Our web collection on statistics for biologists contains articles on many of the points above.

Software and code

Policy information about availability of computer code

Data collection	SerialEM / EPU
Data analysis	cryoSPARC v3.3 / Relion 4.0

For manuscripts utilizing custom algorithms or software that are central to the research but not yet described in published literature, software must be made available to editors and reviewers. We strongly encourage code deposition in a community repository (e.g. GitHub). See the Nature Portfolio guidelines for submitting code & software for further information.

Data

Policy information about availability of data

All manuscripts must include a data availability statement. This statement should provide the following information, where applicable:

- Accession codes, unique identifiers, or web links for publicly available datasets
- A description of any restrictions on data availability
- For clinical datasets or third party data, please ensure that the statement adheres to our policy

The data that support this study are available from the corresponding authors upon request. The coordinates and EM maps generated in this study have been deposited in the Protein Data Bank and the Electron Microscopy Data Bank. All uncropped gels are presented in a source data file.
Accession numbers for new structures determined:
For TiLV polymerase in vRNA initiation state:

(i) PDB ID 8PSN, EMD-17857 (full vRNA initiation with CTP) and
(ii) PDB ID 8PSO, EMD-17858 (core vRNA initiation with CTP).
For TiLV polymerase in cRNA pre-initiation state:
(i) PDB ID 8PSQ, EMD-17860 (core cRNA pre-initiation mode A),
(ii) PDB ID 8PT7, EMD-17869 (core with endo cRNA pre-initiation mode A)
(iii) PDB ID 8PSS, EMD-17861 (closed core with endo cRNA mode B).
For TiLV polymerase in vRNA pre-initiation state:
(i) PDB ID 8PSU, EMD-17862 (core vRNA pre-initiation mode A).
For TiLV polymerase in vRNA elongation state:
(i) PDB ID 8PSX, EMD-17864 (full vRNA elongation with CpNHpp)
(ii) PDB ID 8PSZ, EMD-17865 (full vRNA elongation with CpNHpp and additional mode B promoter).
For TiLV polymerase in vRNA pre-initiation state mode B:
(i) PDB ID 8PT2, EMD-17866 (full with open core vRNA mode B)
(ii) PDB ID 8PTH, EMD-17871 (open core with rotated endo-NLS vRNA mode B)
(iii) PDB ID 8PTJ, EMD-17872 (closed core with rotated endo-NLS vRNA mode B).
For TiLV polymerase in replicase initiation state:
(i) PDB ID 8PT6, EMD-17868 (replicase initiation with CpNHpp).
For TiLV polymerase in the pre-termination state
(i) PDB ID 8QZ8 EMD-18772 (Full vRNA pre-termination with GpCpp)

Accession numbers of previously determined structures referenced in the manuscript
PDB:4WSB, PDB:6QPF, PDB:7QTL

Genbank sequences for the ten genome segments of Tilapia lake virus isolate Til-4-2011, KU751814.1 to KU751823.1

Research involving human participants, their data, or biological material

Policy information about studies with human participants or human data. See also policy information about sex, gender (identity/presentation), and sexual orientation and race, ethnicity and racism.

Reporting on sex and gender	N/A
Reporting on race, ethnicity, or other socially relevant groupings	N/A
Population characteristics	N/A
Recruitment	N/A
Ethics oversight	N/A

Note that full information on the approval of the study protocol must also be provided in the manuscript.

Field-specific reporting

Please select the one below that is the best fit for your research. If you are not sure, read the appropriate sections before making your selection.

[x] Life sciences [ ] Behavioural & social sciences [ ] Ecological, evolutionary & environmental sciences

For a reference copy of the document with all sections, see nature.com/documents/nr-reporting-summary-flat.pdf

Life sciences study design

All studies must disclose on these points even when the disclosure is negative.

Sample size	N/A
Data exclusions	N/A
Replication	N/A
Randomization	N/A
Blinding	N/A

Reporting for specific materials, systems and methods

We require information from authors about some types of materials, experimental systems and methods used in many studies. Here, indicate whether each material, system or method listed is relevant to your study. If you are not sure if a list item applies to your research, read the appropriate section before selecting a response.

Materials & experimental systems

n/a	Involved in the study
☒	☐ Antibodies
☐	☒ Eukaryotic cell lines
☒	☐ Palaeontology and archaeology
☒	☐ Animals and other organisms
☒	☐ Clinical data
☒	☐ Dual use research of concern
☒	☐ Plants

Methods

n/a	Involved in the study
☒	☐ ChIP-seq
☒	☐ Flow cytometry
☒	☐ MRI-based neuroimaging

Eukaryotic cell lines

Policy information about cell lines and Sex and Gender in Research

Cell line source(s)	Trichoplusia ni High5 cells
Authentication	Commerically available cell line from ThermoFisher
Mycoplasma contamination	Not tested for mycoplasma
Commonly misidentified lines (See ICLAC register)	N/A
